# Morphological characterization of breeds of sheep: a discriminant analysis approach

**DOI:** 10.1186/s40064-016-1669-8

**Published:** 2016-01-22

**Authors:** Michael Asamoah-Boaheng, Emmanuel Kofi Sam

**Affiliations:** Institute of Research, Innovation and Development (IRID), Kumasi Polytechnic, Box 854, Kumasi, Ghana; Department of Mathematics, Kwame Nkrumah University of Science and Technology, P.M.B., KNUST, Kumasi, Ghana

**Keywords:** Morphological characterisation, Quadratic discriminant functions, Sheep breeds, Discriminant analysis

## Abstract

In this study the characterisation and separation/discrimination of three sheep breeds (crosses, West African Dwarfs (WAD) and West African Long Legged (WALL)] based on their physical traits (morphological characterisation) was investigated extensively with the application of discriminant analysis. The study’s main objective was specifically based on developing a variable selection criterion that can discriminate best among the three sheep breeds and as well as obtain a reliable mathematical function/equation (discriminant functions) for provision of maximum separation among the three known sheep breeds. Data from College of Education, Mampong animal farms on various breeds of sheep (hybrid/crossed breed, Sahell/WALL and Djallonke/WAD) was used. Factor Analysis was employed as a variable selection criterion for selecting six sheep traits that can discriminate best among the sheep breeds. Canonical discriminant function was derived for the eight variable data set and was compared with the derived quadratic discriminant functions (QDFs) using the six extracted sheep traits. The six variable QDF distance classifier provided maximum separation after cross validation than the 8-variable canonical discriminant functions. The derived mathematical functions (QDFs) were able to provide maximum separation among the three known sheep breeds with a correct classification rate of 0.86.

## Background

The assignment/allocation of individuals/observations to the various known groups with their respective mean vectors and distinguishing characteristics has been a major concern for years and research is ongoing to obtain the best function to ensure maximum separation. This study considered the separation/classification of sheep into their respective groups [cross breed/hybrid West African Longed legged (WALL) and local breed (WAD)] based on their measured physical characteristics by using two classification functions and the evaluation of the performance of the classification functions using error estimators.

Morphological characterization entails the description and documentation of the physical traits of a breed (Rege 1992). The *World Watch List for Domestic Animal Diversity* (*WWL-DAD*) prepared by FAO (2000) defined a breed as either a homogenous, sub-specific group of domestic livestock with definable and identifiable external characteristics that enable it to be separated by visual appraisal from other similarly defined groups within the same species.

Characterization of animal genetic resource (AnGR) encompasses all activities associated with the identification, quantitative and qualitative description of breed populations and the natural habitat and production systems to which they are or not adapted. Food and Agricultural Organisation FAO (2007) estimates that industrial livestock operations are growing twice as fast as traditional mixed farming systems and six times as fast as traditional grazing systems. Sheep seem to have received the least attention in all aspect of management, nutrition breeding and health in spite of the fact that they have many merits over some other classes of livestock and are found in all towns and villages in Ghana (Koney 2004). In Ghana, sheep are often seem to roam about to fend for themselves during the day in many rural areas with animals from different households mixing together of unknown records. Livestock production is a major feature in Ghana’s agriculture and contributes largely towards meetings food needs, providing drought power, manure to maintain soil fertility and structure and cash income, particularly for farmers in the northern part of the country (Oppong-Anane 2006).

Discriminant analysis is used in situations where the clusters are known a priori. The aim of discriminant analysis is to classify an observation, or several observations, into already known groups (Hardel and Simar 2007). The problem of statistical discrimination involving three multivariate normal distributions with known or unknown population centroids and with equal (or unequal) covariance matrices has been considered by many researchers. Some other researchers have applied the concept of discriminant analysis which also serves as a classificatory rule in allocating observations/objects into their known groups. Researchers including Fisher (1936), Lachenbruch (1975), Krzanowski and Hand (1997), Desu and Geisser (1973) have used discriminant analysis extensively in various fields where mostly linear discriminant function (LDF) was the main classification function obtained for classifying the known observations.

A research paper published in the International Journal of Biodiversity and Conservation, volume 5 on Morphometric characterization of Nigerian indigenous sheep using multifactorial discriminant analysis was investigated by Yunusa et al. (2013). Stepwise multifactorial discriminant analysis was employed. Among the eight (8) distinguishing traits found, their length of tail was found to be the most discriminating character. A multivariate analysis of phenotype differentiation in Bunaji and Sokoto Gudali cattle was investigated by Yakubu et al. (2010b). The researcher applied multi-factorial discriminant analyses using ten morphological traits in examining morphometric differentiation in two Nigerian breeds of cattle. The Nearest Neighbour Discriminant Analysis was employed and 85.48 % Bunaji cattle were classified into their source population while 96.55 % of their Sokoto Gudali counterparts were correctly assigned into their source genetic group.

Herrera et al. (1996) studied an application of a multifactorial discriminant analysis in the morpho-structural differentiation of Andalusian caprine breeds in Spain. Yakubu et al. (2010a) conducted a study by applying the concept of discriminant analysis on morphometric differentiation in West African Dwarf and Red Sokoto goats. Aziz and Al-Hur (2013) applied Size-free Canonical Discriminant Analysis in differentiating between three Saudi goat types. They utilised body weight and 16 body measurements randomly selected from the three Saudi goats and was used to discriminate between 188 animals after conducting a size free discriminant analysis on the data.

Traore et al. (2008) investigated into multivariate characterization of morphological traits in Burkina Faso sheep. Their study was based on 6440 female sheep from Burkina Faso and seven body measurements were taken as well as four qualitative morphological traits. Their study sample also included three main environmental areas and sheep breeds of Burkina Faso namely the Sahel area (Burkina-Sahel sheep), the Sudan-Sahel area (Mossi sheep) and the Sudan area (Djallonke sheep). Results from the Canonical analysis showed that, there exist small differences in the recorded body measurements of Sudan and the Sudan-Sahel sheep even though most body traits showed higher average values in the Burkina-Sahel sheep. Ebegbulem et al. (2011) researched into the morphometric differentiation of West African Dwarf Goats in southeastern Nigeria using discriminant Analysis. One hundred and twenty-one (121) West African Dwarf (WAD) goats aged between <1 year and 4 years sampled from local farmers from Nigeria were used for the study. After the application of discriminant analysis, 83.5 % of correct classification of goats was achieved.

## Methods

This part of the study explains in details the various methods employed in the analysis of the data. The method of analysis looks at discriminant analysis approach in general and factor analysis as a criterion for variable selection (i.e. data reduction tool).

### Data used

The data used was based on 61 sheep breeds which comprises the crosses, the Djallonke and the West African Longed Legged (WAD) breeds with eight measured morphological traits namely Height at withers (Ht), Body Length (Lt), Ear Length (EL), Weight (Wt), Chest girth (Chst), Hook Length (HL), Tail Length (TL) and Difference between Hook length and Tail length (HL-TL) which was collected from the College of Science Animal farm at Mampong Ashanti.

### Allocation rules for known distributions

Discriminant analysis is a set of methods and tools used to distinguish between groups of populations, $\pi_{i}$ and to determine how to allocate new observations into groups. In general we have populations $\pi_{j} ,j = 1,2, \ldots ,J$ and we have to allocate an observation *x* to one of these groups.

### Classification with equal covariance matrices ($\Sigma_{i} = \Sigma_{j} = \Sigma$)

The density of population $\pi_{i}$, $i = 1,2$ is given by;

1 $$f_{i} (x) = \frac{1}{{(2\pi )^{p/2} |\Sigma |^{1/2} }}\exp \left( { - \frac{1}{2}(x - \mu_{i} )^{'} \Sigma^{ - 1} (x - \mu_{i} )} \right)$$

If the populations $\pi_{1}$ and $\pi_{2}$ both have multivariate normal densities with equal covariance matrices, then the classification rule corresponding to minimizing Expected Cost of Misclassification (ECM) becomes:

Classify $x_{0}$ as $\pi_{1}$ if

2 $$(\mu_{1} - \mu_{2} )^{'} \Sigma^{ - 1} x_{0} - \frac{1}{2}(\mu_{1} - \mu_{2} )^{'} \Sigma^{ - 1} (\mu_{1} + \mu_{2} ) \ge \left( {\ln \left( {\frac{c(1|2)}{c(2|1)}} \right)\left( {\frac{{p_{2} }}{{p_{1} }}} \right)} \right)$$

The sample estimates for Eq. (2) can be obtained by replacing $\mu_{1}$, $\mu_{2}$ and $\Sigma$ with $\bar{x}_{1}$, $\bar{x}_{2}$ and $S_{pooled}$. For a special case for Eq. (2), when the prior probabilities and the misclassification cost are equal, we assign $x_{0}$ to $\pi_{1}$ if:

3 $$(\bar{x}_{1} - \bar{x}_{2} )^{'} S_{pooled}^{ - 1} x_{0} \ge \frac{1}{2}(\bar{x}_{1} - \bar{x}_{2} )^{'} S_{pooled}^{ - 1} (\bar{x}_{1} + \bar{x}_{2} )$$

Denote $a = S_{pooled}^{ - 1} (\bar{x}_{1} - \bar{x}_{2} ) \in \Re$ and the above equation can be written as

4 $$a^{'} x_{0} \ge \frac{1}{2}\left( {a^{'} \bar{x}_{1} + a^{'} \bar{x}_{2} } \right)$$

(Johnson and Wichern 2007). Similar approach was applied when three populations were considered in this study.

### The quadratic classifier $\left( \sum_{1} \ne \sum_{2}\right)$

The regions of minimum ECM and minimum total probability of misclassification (TPM) depends on the ratio of the densities. Hence substituting the normal densities with different covariance matrices in Eq. 1 after taking natural logarithm gives the following classification regions. Allocate *x* to $\pi_{1}$ or otherwise to $\pi_{2}$ if,

5 $$- \frac{1}{2}x^{'} \left( {S_{1}^{ - 1} - S_{2}^{ - 1} } \right)x + \left( {\bar{x}_{1}^{'} S_{1}^{ - 1} - \bar{x}_{1}^{'} S_{2}^{ - 1} } \right)x - k \ge \ln \left[ {\left( {\frac{c(1|2)}{c(2|1)}} \right)\left( {\frac{{p_{2} }}{{p_{1} }}} \right)} \right]$$

where

$$k = \frac{1}{2}\ln \left( {\frac{{|S_{1} |}}{{|S_{2} |}}} \right) + \frac{1}{2}\left( {\bar{x}_{1}^{'} S_{1}^{ - 1} \bar{x}_{1}^{'} - \bar{x}_{2}^{'} S_{2}^{ - 1} \bar{x}_{2} } \right)$$

### Classification into several populations

Generalization of classification procedure for more than two discriminating groups (i.e. from 2 to $g \ge 2$) is straight forward. However, not much is known about the properties of the corresponding sample classification function, and in particular, their error rates have not been fully investigated. Therefore, we focus only on the Minimum ECM Classification with equal misclassification cost and Minimum Total Probability of Misclassification (TPM) for multivariate normal population with unequal covariance matrices (Quadratic discriminant analysis).

### Cross validation (CV)

The (leave-one-out) cross-validation or jackknife procedure or the Holdout method which works as follows:

1. Leave one object out of the sample and construct a classification rule based on the remaining $n - 1$ objects in the sample.
2. Classify the left-out observation using the classification rule obtained in step 1 above.
3. Repeat the two previous steps for each of the objects in the sample.
4. Let $n_{1M}^{CV}$ and $n_{2M}^{CV}$ be the number of left out observations misclassified in group 1 and 2 respectively and it’s given by
  6 $$CV = \frac{{n_{1M}^{CV} + n_{2M}^{CV} }}{{n_{1} + n_{2} }}$$

(Johnson and Wichern 2007).

### Factor analysis (FA) as a variable selection criterion

The major aim of factor analysis is the orderly simplification of a large number of intercorrelated measures to a few representative constructs or factors. The primary function of factor analysis is to identify these clusters of high inter-correlations as independent factors. The main steps involved in factor analysis are; computation of the correlation matrix, extraction of initial factors, determining the number of factor’s to be extracted and rotation methods.

#### Orthogonal factor model

The aim of factor analysis is to explain the outcome of *p* variables in the data matrix *X* using fewer variables (i.e. the so-called factors). Ideally all the information in *X* can be reproduced by a smaller number of factors. These factors are interpreted as latent (unobserved) common characteristics of the observed $x \in \Re^{p}$. The case just described occurs when every observed $x = (x_{1} , \ldots ,x^{p} )^{'}$ can be written as

7 $$X_{j} = \sum\limits_{l}^{k} {q_{jl} f_{l} + \mu_{j} ,\quad j = 1, \ldots ,p}$$

where $f_{l} ,l = 1, \ldots ,k$, denotes the factors, $q_{jl}$ is the loading of the *jth* variable on the *lth* factor, $\mu_{j}$ is the mean of the variable *j*. It is therefore expected that, the number of factors *k* should always be much smaller than *p* (Hardel and Simar 2007).

## Results

This part of the study presents the results of the study as well as extensive discussion.

### Preliminary findings

The various traits/characteristics of the various sheep breeds considered were their *Height (Ht)*, *Length (Lt),Ear Length (EL)*, *Weight (Wt)*, *Chest Size (Chst)*, *Hook Length (HL)*, *Tail Length (TL)* and *Difference between Hook length and Tail length (HL*-*TL)*. Preliminary findings based on their computed means and their respective standard deviations shows some differences in the measured traits across the three breeds (see Table 1). Test of significance was conducted to test statistically whether there are differences among the group means of the measured traits for the various breeds of sheep. F-test conducted indicated significant differences between the mean measured traits for the three sheep breeds.

**Table 1:** Descriptive statistics of the data

	Breed type			Total
	Djallonke	Sahel	Crosses	
*Mean (traits)*				
Height	52.69570	62.55560	52.68970	54.15000
Length (Lt)	54.04348	57.88889	53.86207	54.52459
Ear length (EL)	9.304348	12.22222	9.517241	9.836066
Tail length (TL)	24.52170	33.22220	26.72410	26.85000
Weight (Wt)	20.08696	23.44444	20.79310	20.91803
Chest (chst)	67.08696	69.55556	66.48276	67.16393
Hook length (Hk lgth)	27.56522	30.11111	27.10345	27.72131
Hook and tail length (Hk lth-Tlth)	3.086957	−3.11111	0.275862	0.836066
*SD (traits)*				
Height	4.14986	9.22105	4.90702	6.429
Length (Lt)	4.72400	7.49074	5.84761	5.798
Ear length (EL)	0.70290	1.39443	0.68768	1.293
Tail length (TL)	2.74474	3.52767	3.33698	4.218
Weight (Wt)	4.93515	10.38161	7.15332	6.958
Chest (chst)	5.47650	10.93288	8.41181	7.813
Hook length (Hk lgth)	1.97314	4.88478	2.80745	3.056
Hook and tail length (Hk lth-Tlth)	2.04302	2.36878	1.99815	2.928

First the equality of the three covariance matrices were tested with Box M test of equality of covariance matrices of the three sheep breeds under study. The log determinants of the three covariance matrices for two groups were found from the table as almost equal with the other one slightly apart from the other two (see Table 2). The hypothesis for testing the equality of covariance matrices was stated as:

**Table 2:** Test for equality of covariance matrices

Log determinants		Log determinant	Test results	
Breed type	Rank			54.84
Djallonke/WAD	8	6.742	Approx.	1.256
Sahel/WALL	7		Df1	36
Crosses	8	10.582	Df2	7506.655
Pooled within-groups	8	10.597	P value	0.141

$H_{0} :\sum_{1} = \Sigma_{2} = \Sigma_{3}$ Vrs $H_{1} :$*At least one pair of Sigma’s (*$\Sigma$*) is different*.

From Box M table, we observed a *p* value of 0.141 and since the observed *pvalue* is greater than the significance ($\alpha$) level of 5 %, we fail to reject the null hypothesis of no difference and conclude that, all the three covariance matrices are equal. Based on these results, all discriminant/classification functions will assume a linear approach.

### Canonical linear discriminant function (FLDF) for classification

Canonical linear discriminant function (FLDF) was employed using all the eight variate data set after testing for equality of the covariance matrices among the three sheep breeds. The Box M test above as shown in Table 2 was insignificant and hence a linear function was appropriate for classification. An eight variable canonical discriminant functions were derived based on the fact that, the major assumption of discriminant analysis was not violated (equal covariance matrices across the three groups).

First and foremost, in order to determine whether the functions to be derived are significant or not, there is the need for the researcher to know the number of functions needed for the separation purposes. Hence the number of functions equals the number of groups/sheep breeds minus one. In this case, we have three groups (WAD, WALL and hybrid/crosses), thus we have 3 − 1 = 2 possible functions needed for separation purposes. This is evident in Tables 3 and 4 where the first function (function 1) explaining 93.1 % of the variance and has a small lambda (0.166) and it’s significant with *pvalue* of 0.000. The second function explains only 6.9 % of the variance in the data, with a recorded *pvalue* of 0.066. Therefore, the second function does not contribute much significantly in the discrimination process as compared to that of the first function. In other words, this factor does not help much in discriminating the groups.

**Table 3:** Table of eigenvalues

Function	Eigenvalue	% of variance	Cumulative %	Canonical correlation
1	3.734	93.1	93.1	0.888
2	0.275	6.9	100.0	0.465

**Table 4:** Wilks lambda test

Function	Wilks’ lambda	Chi square	Df	P value
1 through 2	0.166	97.987	16	0.000
2	0.784	13.256	7	0.066

In conducting discriminant analysis, the entire data was standardised due to different measurement scales used for the various breed traits to assume a unit variance or dispersion, under the standard normal distribution. The two derived canonical discriminant functions are

8 $$DF_{1} = 9.56 - 0.11Ht - 0.07Lt - 0.88EL + 0.37TL - 0.04Wt + 0.13Chst - 0.35HL + 0.71(HL - TL)$$

9 $$DF_{2} = - 6.74 + 0.14Ht - 0.031Lt - 0.23EL + 1.63TL - 0.07Wt - 0.01Chst - 1.61HL + 1.93(HL - TL)$$

After computing the discriminant scores using the above two equations, the following proportion of correct classification and misclassifications were recorded and are presented in Table 5. Observations were classified into their desired group under unequal group prior probabilities.

**Table 5:** Classification results of the eight variate data

	Breed type	Predicted group membership			Total
		Djallonke/WAD	Sahel/WALL	Crosses	
*Original*					
N	Djallonke/WAD	15	0	8	23
	Sahel/WALL	0	8	1	9
	Crosses	5	0	24	29
%	Djallonke/WAD	65.2	0	34.8	100.0
	Sahel/WALL	0	88.9	11.1	100.0
	Crosses	17.2	0	82.8	100.0
*Cross-validated*					
N	Djallonke/WAD	15	0	8	23
	Sahel/WALL	0	8	1	9
	Crosses	6	0	23	29
%	Djallonke/WAD	65.2	0	34.8	100.0
	Sahel/WALL	0	88.9	11.1	100.0
	Crosses	20.7	0	79.3	100.0

From Table 5, 65.2 % of the original observations from the Djallonke/WAD sheep group were correctly classified, with the remaining 34.8 % being misclassified into the sheep crosses group. Also 88.9 % of the Sahel/WALL sheep breeds were correctly classified into their respective group, only one (1) representing 11.1 % being misclassified into the crosses sheep breed. The functions derived were able to separate the cross sheep breed form the other breeds with 82.8 % correct classification of the cross breed into their desired group with the remaining 17.2 % being misclassified into the Djallonke/WAD sheep breed. In all, approximately 77.0 % correct classification of the sheep breeds using the linear discriminant functions with eight variables/traits was achieved. Also the correct classification rate for the cross validated results was 75.4 %.

### A six variable discriminant function using quadratic discriminant function (QDF)

Factor analysis was employed as a variable selection criterion for selecting the major variables/traits for the provision of maximum separation among the three known sheep breeds. All the four main steps in factor analysis were followed and out of the eight morphological traits, six traits including Length (Lt), Ear length (EL), Weight (Wt), Chest (Wt), Hook Length (HL), Hook Length and Tail Length (HL-TL) were extracted after VARIMAX rotation method as shown in Table 6.

**Table 6:** VARIMAX rotated component matrix under factor analysis

Traits/physical characteristics	Factor	
	1	2
*Height (Ht)*	*0.799*	*0.445*
Length (Lt)	0.906	
Ear Length (EL)		0.757
*Tail length (TL)*	*0.492*	*0.821*
Weight (Wt)	0.895	
Chest (chst)	0.947	
Hook length (Hk lgth)	0.788	
Hook and tail length (Hk lth-Tlth)		−0.866

The *italic* observations indicate cross loaded observations and by rule they are discarded

In checking the equality of the covariance matrices for the three groups using the new data (six variate data), Box M test was employed and the three covariance matrices of the sheep breeds were found to be unequal or at least one of the covariance matrices is not equal to the other. Hence, since the covariance matrices are not equal, the appropriate discriminant function to be derived for classification of the sheep breeds using the six variate data is the Quadratic Discriminant Function (QDF).

In this case, two discriminant functions were derived to classify the sheep breeds into their respective groups under unequal prior probability and equal misclassification cost. The two functions derived are as follows;

10 $$(x - \bar{x}_{1} )^{'} S_{1}^{ - 1} (x - \bar{x}_{1} ) - (x - \bar{x}_{2} )^{'} S_{2}^{ - 1} (x - \bar{x}_{2} ) \le 0.377$$

11 $$(x - \bar{x}_{1} )^{'} S_{1}^{ - 1} (x - \bar{x}_{1} ) - (x - \bar{x}_{3} )^{'} S_{3}^{ - 1} (x - \bar{x}_{3} ) \le 0.148$$

Based on the above quadratic discriminant functions, the various probabilities of correct classifications and misclassifications were obtained and are presented in Table 7.

**Table 7:** Probabilities of correct classifications and misclassifications

Observations	Classification		Probabilities		
	TRUE	Class.	Djallonke/WAD	Sahel/WALL	Crosses
1	Sahel/WAD	Sahel/WAD	0	1	0
2	Sahel/WAD	Sahel/WAD	0	1	0
3	Crosses	Crosses	0.0111	0.0013	0.9876
4	Sahel/WAD	Sahel/WAD	0	1	0
5	Crosses	Crosses	0.0727	0.1946	0.7327
6	Crosses	Crosses	0.0396	0	0.9604
7	Crosses	Crosses	0.0989	0.0012	0.8999
8	Sahel/WAD	Sahel/WAD	0	1	0
9	Sahel/WAD	Sahel/WAD	0	1	0
10	Crosses	Crosses	0	0	1
11	Sahel/WAD	Sahel/WAD	0	0.9998	0.0001
12	Sahel/WAD	Sahel/WAD	0	1	0
13	Crosses	Crosses	0.0155	0	0.9845
14	Sahel/WAD	Sahel/WAD	0	1	0
15	Sahel/WAD	Sahel/WAD	0.0567	0.6766	0.2667
16	Crosses	Crosses	0.2552	0	0.7448
17	Crosses	Crosses	0.409	0	0.591
18	Crosses	Crosses	0.3286	0.0003	0.6711
19	Crosses	Crosses	0.2602	0	0.7398
20	Crosses	Crosses	0.4336	0	0.5664
21	Crosses	Crosses	0.268	0	0.732
22	Crosses	Crosses	0.0426	0	0.9574
23	Crosses	Crosses	0.2697	0	0.7303
24	Crosses	Crosses	0	0	1
25	Crosses	Crosses	0.4503	0	0.5497
26	Crosses	Crosses	0.0774	0	0.9226
27	Djallonke	Crosses*	0.4958	0	0.5042
28	Crosses	Crosses	0.0926	0	0.9074
29	Djallonke	Crosses*	0.439	0	0.561
30	Djallonke	Djallonke	0.6132	0	0.3868
31	Djallonke	Djallonke	0.6708	0	0.3292
32	Crosses	Crosses	0.2863	0	0.7137
33	Crosses	Djallonke*	0.5045	0	0.4954
34	Djallonke	Crosses*	0.4141	0	0.5859
35	Crosses	Crosses	0.4416	0	0.5584
36	Crosses	Djallonke*	0.627	0	0.373
37	Djallonke	Djallonke	0.5014	0	0.4986
38	Djallonke	Crosses*	0.1932	0	0.8068
39	Djallonke	Djallonke	0.9076	0	0.0924
40	Djallonke	Djallonke	0.7433	0	0.2567
41	Djallonke	Djallonke	0.9266	0	0.0734
42	Djallonke	Djallonke	0.5118	0	0.4882
43	Djallonke	Djallonke	0.9428	0	0.0572
44	Djallonke	Djallonke	0.7214	0	0.2786
45	Crosses	Djallonke*	0.6205	0	0.3795
46	Crosses	Crosses	0	0	1
47	Crosses	Djallonke*	0.875	0	0.125
48	Crosses	Djallonke*	0.6798	0	0.3202
49	Crosses	Djallonke*	0.72	0	0.28
50	Djallonke	Djallonke	0.9915	0	0.0085
51	Crosses	Djallonke*	0.5377	0	0.4623
52	Crosses	Crosses	0.365	0	0.635
53	Djallonke	Djallonke	0.9978	0	0.0022
54	Djallonke	Djallonke	0.8449	0	0.1551
55	Djallonke	Djallonke	0.7136	0	0.2864
56	Djallonke	Djallonke	0.9022	0	0.0978
57	Djallonke	Djallonke	0.9561	0	0.0439
58	Djallonke	Djallonke	0.9207	0	0.0793
59	Djallonke	Djallonke	0.9989	0	0.0011
60	Djallonke	Djallonke	0.9993	0	0.0007
61	Djallonke	Djallonke	0.9988	0	0.0012

* Misclassified observations

From Table 7, three (3) Djallonke sheep breeds were misclassified into the cross sheep breeds, six (6) observations were also misclassified from the cross breed to Djallonke sheep breed. In all nine (9) sheep breeds were misclassified from either Djallonke or crosses sheep breed. None of the Sahel/WAD sheep breeds were misclassified into either Djallonke or crosses breed. In summary, out of the total sixty-one (61) sheep breeds, 52 of them were correctly classified into their respective sheep breed representing approximately 85 % with only nine being misclassified. The summaries of classification and misclassification rates are presented in the confusion matrix table as shown in Table 8.

**Table 8:** Confusion matrix for summary of classification of the six variate data

	Classified			Total
True	Djallonke/WAD	Sahel/WALL	Crosses	
*Original*				
Djallonke/WAD	19 82.61	0 0.00	4 17.39	23 100.00
Sahel/WALL	0 0	9 100	0 0	9 100.00
Crosses	7 24.14	0 0.00	22 75.86	29 100.00
Total	26 42.62	9 14.75	26 42.62	61 100.00
Priors	0.3770	0.1475	0.4754	
*Cross-validated/leave-one-out*				
Djallonke/WAD	20 86.95 %	0	3 13.05 %	23 100.0 %
Sahel/WALL	0	9 100.0 %	0	9 100.0 %
Crosses	5 17.24 %	0	24 82.76 %	29 100.0 %

From Table 8, 82.61 % of correct classifications of Djallonke/WAD sheep breeds were recorded, with a misclassification rate of 0.1739 into the crosses sheep breed. Also none of the Sahel/WALL sheep breeds were misclassified and a 100 % correct classification was achieved. For the crosses breed, results from Table 8 shows 75.86 correct classification with only 24.14 % of them being misclassified into the Djallonke/WAD sheep breed. The table also summarises the results of cross validated results. In all, approximately 82.0 % correct classification of sheep breed was achieved under classification with QDF as well as 86.9 % correct classification rate under the cross validated results. This study therefore conforms with the research based study by Traore’ et al. (2008), Aziz and Al-Hur (2013), Yakubu et al. (2010b), Ebegbulem et al. (2011) and Agavierzor et al. (2012) where all these researchers applied discriminant analysis in separating the known breeds of animals using significant morphological traits as the main variables for maximizing separation.

## Conclusion

The study was aimed at establishing a separator/discriminating function for separating the three known sheep breeds (hybrid/crosses, WAD and WALL sheep breeds). The derived discriminant functions provided maximum (canonical linear discriminant function) separation among the three known breeds with an overall classification rate of 78.9 %. However, factor analysis extracted six (6) traits out of the eight variables and the derived discriminant functions with the six variables provided better separation than the eight variate discriminant equation (Canonical discriminant function). Quadratic discriminant functions were derived from the six variate data and 86.2 % correct classification of sheep breeds were achieved. The study can therefore conclude that sheep breeds can be clearly separated based on the physical traits with minimum rate of misclassification without concentrating on only their genotypic features.
